# Fires in Seasonally Dry Tropical Forest: Testing the Varying Constraints Hypothesis across a Regional Rainfall Gradient

**DOI:** 10.1371/journal.pone.0159691

**Published:** 2016-07-21

**Authors:** Nandita Mondal, Raman Sukumar

**Affiliations:** 1 Centre for Ecological Sciences, Indian Institute of Science, Bangalore—560012, Karnataka, India; 2 Divecha Centre for Climate Change, Indian Institute of Science, Bangalore—560012, Karnataka, India; Ecole Pratique des Hautes Etudes, FRANCE

## Abstract

The “varying constraints hypothesis” of fire in natural ecosystems postulates that the extent of fire in an ecosystem would differ according to the relative contribution of fuel load and fuel moisture available, factors that vary globally along a spatial gradient of climatic conditions. We examined if the globally widespread seasonally dry tropical forests (SDTFs) can be placed as a single entity in this framework by analyzing environmental influences on fire extent in a structurally diverse SDTF landscape in the Western Ghats of southern India, representative of similar forests in monsoonal south and southeast Asia. We used logistic regression to model fire extent with factors that represent fuel load and fuel moisture at two levels—the overall landscape and within four defined moisture regimes (between 700 and1700 mm yr^-1^)—using a dataset of area burnt and seasonal rainfall from 1990 to 2010. The landscape scale model showed that the extent of fire in a given year within this SDTF is dependent on the combined interaction of seasonal rainfall and extent burnt the previous year. Within individual moisture regimes the relative contribution of these factors to the annual extent burnt varied—early dry season rainfall (i.e., fuel moisture) was the predominant factor in the wettest regime, while wet season rainfall (i.e., fuel load) had a large influence on fire extent in the driest regime. Thus, the diverse structural vegetation types associated with SDTFs across a wide range of rainfall regimes would have to be examined at finer regional or local scales to understand the specific environmental drivers of fire. Our results could be extended to investigating fire-climate relationships in STDFs of monsoonal Asia.

## Introduction

Fires in natural ecosystems are a global phenomenon, occurring in almost every ecosystem type across the globe [1]. However, the extent to which fire spreads depends largely on the kind and amount of fuel available for burning as well as the moisture content of the fuel. Climate is the overarching factor that influences fuel type, amount and moisture content through its influence on the type of vegetation, productivity, biomass decomposition and its state of desiccation in an area [2–4]. However, there has been much discussion on whether the amount of potential fuel or its moisture content is the driving factor for fire behavior and spread in an ecosystem [5].

In a synthesis of studies on large, infrequent fire events at local and regional scales the authors hypothesized that the relative contribution of fuel load and fuel moisture to fire in various ecosystems differs across regions globally [6]. They outlined a conceptual global model for large fires in which fuel moisture and fuel amount are “the endpoints of a gradient…determined by long-term climatic conditions”. A particular ecosystem would lie at a point along this gradient depending on the relative contribution of these two factors to the fire event. This concept was extended in another study to general global fire activity [7]. Their analysis of fire events with soil moisture metrics and tropospheric circulation quantifies this variation in fuel amount and fuel moisture over a global scale, validating the global conceptual model they term as the “varying constraints hypothesis”.

The global concept of the varying constraints hypothesis can also be examined at condensed spatial scales in regions with distinct rainfall gradients where variations in fuel load and moisture may result in differences in the extent of fire. Although not explicitly testing this hypothesis, several regional studies have examined the relationship of fire extent with fuel conditions along such rainfall gradients [8–11]. These studies have found a unimodal relationship of area burnt with increasing rainfall–areas with high and low rainfall have low area burnt, whereas areas with intermediate rainfall have the highest area burnt.

Seasonally dry tropical forests (SDTFs), rather than being composed of a single physiognomic type, encompass a range of vegetation types depending upon the rainfall regime that could range from 250mm to 2000mm yr^-1^ [12]. Characteristically, SDTFs have a defined dry season that could extend up to 8 months [12] with mean precipitation per month less than 100mm. This seasonality makes these forests susceptible to fire almost every year [13], unlike the very moist tropical rainforest or evergreen forests that are susceptible to fire under the most extreme conditions of moisture deprivation due to drought [6,14,15]. Indeed, fire is a major determinant of the structure and diversity of SDTFs [16,17]. The combination of rainfall regimes as well as seasonality within a year provides an interesting framework for testing the varying constraints hypothesis as it relates to fire within SDTFs. At the same time, SDTFs have not been placed as a unique entity in the context of the global fire hypothesis. Biomes that comprise SDTFs such as savannas and tropical/subtropical broadleaf deciduous forest have been placed along the global gradient as separate entities, with fires in broadleaf deciduous forest being more moisture limited as compared to savannas [7].

South and south-east Asia have extensive areas under SDTFs that experience a monsoonal climate [18]. The few studies on fire ecology in SDTFs of this region have characterised fires and fire regimes in these forests [17,19–21], but have not analysed the environmental constraints on fires, perhaps because of the general absence or paucity of detailed, long-term climate and fire data specific to such forests. We attempt to fill this gap by analysing a 2-decade long data set on rainfall and fire in an SDTF at Mudumalai that is representative of a range of forest vegetation types in southern peninsular India occurring along a rainfall gradient–from tropical moist deciduous forest in the Western Ghats to dry thorn forest in the Eastern Ghats [22, 23]. The fire-climate patterns from this region would also be broadly applicable to SDTFs in monsoonal south and southeast Asia.

We address two aspects of fire in this southern Indian SDTF with respect to the global varying constraints hypothesis. Our first objective is to place this SDTF within the context of the global hypothesis. Specifically we ask the question as to whether fires in SDTFs are limited by fuel amount or fuel moisture. The second objective is to test the applicability of the varying constraints hypothesis to an SDTF that exhibits variations in rainfall and forest structure across the landscape and, hence, variations in fuel amount and fuel moisture. Our hypothesis is that the relative influence of fuel load and fuel moisture would vary across the rainfall gradient, with fires in the wetter portions of the study area being more limited by fuel moisture, whereas fuel load would limit fires in the driest areas.

## Methods

This study was carried out in Mudumalai Wildlife Sanctuary and National Park (c. 321 km^2^, henceforth Mudumalai) located in the state of Tamil Nadu, southern India (N 11°32.0'–11°42.3', E 76°21.4'–76°44.9'). Permissions to conduct research in Mudumalai were obtained from the Tamil Nadu Forest Department, Tamil Nadu, India. The elevation within Mudumalai ranges from 485m to 1266m above mean sea level (asl) with 95% of the area lying between 800m and 1100m asl (Figure A in S1 Appendix). The topography of the Western Ghats to the west [24] and the Nilgiri massif to the southeast of Mudumalai (Figure A in S1 Appendix) influences the distribution of rainfall across the Mudumalai landscape, with higher rainfall in the west as compared to the east. The tropical vegetation type across Mudumalai corresponds to this rainfall gradient with moist deciduous and patches of semi-evergreen forest in the south-west, dry deciduous and dry dipterocarp forests over the central part, and dry thorn forest in the east. Swampy grasslands, most of which are now cultivated, also occur in the western part of the landscape. Floristic descriptions for each forest type of Mudumalai are available elsewhere [25]. Canopy cover varies from about 80% in the moist deciduous forest through 50% in the dry deciduous forest to a low of 20% in the dry thorn forest [17, 26]. The fairly closed canopy in the semi-evergreen forest type restricts the growth of grasses. The more open canopy in dry and degraded moist deciduous parts promotes the growth of tall, perennial grasses while shorter grasses are common in the dry thorn forest [17, 25]. Trees begin to shed their leaves during the dry season beginning in December, and canopies are typically completely bare by February. Grasses and leaf litter make up the primary fuel components for the predominantly surface fires in the study area. The average fire return interval for Mudumalai for 1989–2002 was reported as 3.3 years, with dry deciduous forest and dry dipterocarp forest having the shortest intervals (1.7 and 2.9 years respectively), and dry thorn forest and moist deciduous forest having the longest intervals (6.8 and 5.5 years respectively) [21].

### Area burnt

Most fires at Mudumalai occur during January-April with a peak in February-March, corresponding to the main dry season. The area affected by fire at Mudumalai has been annually mapped by our research team through ground surveys from 1989 to 2002, by using GPS from 2003 to 2010, and digitised in a GIS domain. These field surveys have been supplemented by satellite imagery for the years 1996, 1997, 1999, 2001, 2002 and 2005 [17] and were used in the analysis after verification with the ground truth maps (see ‘Note on area burnt data’ and Table A in S3 Appendix). Area burnt was totalled for each year and each rainfall regime using Universal Transverse Mercator (UTM) projection (UTM zone43, datum: WGS 84) (Figure B in S1 Appendix).

### Moisture regimes

Average annual rainfall for the period 1990–2010 was derived from each of 13 rain gauges within and close to Mudumalai. This was used to derive a map of average annual rainfall over the landscape at 100m × 100m resolution by kriging [27]. The map was then used to derive moisture regimes based on rainfall contours at 200mm interval. Four moisture regimes (MR) were defined for the study area as follows–MR1: ≥ 1400 mm yr^-1^; MR2: 1200–1400 mm yr^-1^; MR3: 1000–1200 mm yr^-1^; MR4: ≤ 1000 mm yr^-1^ (Figure B in S1 Appendix). These roughly correspond to the distribution of moist deciduous/semi-evergreen forest, degraded moist deciduous/dry deciduous forest, dry dipterocarp/dry deciduous forest, and dry thorn forest, respectively.

### Seasonal rainfall

The wet season typically begins with convectional rains in the months of April and May termed as the “pre-monsoon” period; the contribution of rains in May to annual rainfall can be significant [28, 29]. This is followed by the summer southwest monsoon during June-September that brings approximately 60% of the annual rainfall to Mudumalai. The winter northeast monsoon sets in by October and can last until early December; the rains during this phase typically occurs in short, heavy spells, and are highly variable especially in November.

High rainfall in the previous year’s wet season is usually taken as a measure of increased productivity and, therefore, high fuel load in the fire season the following year [4, 7]. The length of the dry season [11] or rainfall received in the dry season [4] is taken as a factor influencing the drying of fuels. We defined two seasonal rainfall categories: (a) Wet season rainfall of the previous year–as an indicator of productivity/fuel load. This is the sum of rainfall in the months from May to October the year before the fire season. (b) Early dry season rainfall–as an indicator of fuel moisture. This is the rainfall during November and December; fuels would dry out in the early part of the dry season if the monsoon withdraws early (Figure C in S1 Appendix). Rainfall data that were missing for a few months/years at some stations (c. 11% for both early dry season and wet season rainfall) were imputed from linear regressions of rainfall data of the target station with other stations in the dataset. Maps were generated at 100m × 100m resolution for seasons in each year. Since wet season rainfall was spatially autocorrelated between stations, universal kriging with linear drift was used for the interpolation of wet season rainfall. There were no similarities in rainfall data between neighbouring stations in the case of the early dry season rainfall data; hence, inverse distance weighting was used to interpolate rainfall for the early dry season. The non-similarity could be because rainfall during the early dry season is mostly convectional that is locally restricted and unpredictable.

Wet season rainfall was positively related to fuel load measurements taken at 87 locations across the landscape of Mudumalai during 2004–2006 (Figures A and C in S2 Appendix); thus, the use of wet season rainfall as a proxy for fuel load available for burning the following year was justified. Wet season rainfall and early dry season rainfall for each of the 87 points were extracted from interpolated maps for the years 1990–2009 in order to provide a representative sample of points for rainfall across the sanctuary. Seasonal rainfall for a year was represented as the spatial average of rainfall extracted from all 87 point locations for a year.

### Statistical analyses

Visual examination of data from the Mudumalai landscape of area burnt in a year with the previous year’s wet season rainfall and early dry season rainfall (Figure A in S3 Appendix) suggested that the former could be influenced by interactions between these seasonal rainfall measures as well as area burnt the previous year. Proportion area burnt in a year for the landscape was therefore modelled as a function of a 3-way interaction between wet season rainfall the previous year, early dry season rainfall and proportion area burnt the previous year. This analysis addresses the first objective–placing SDTFs within the context of the global gradient. Generalised linear models (GLMs) with quasi-binomial error structure and logit link function were used in the analysis. The logit link function limits the predicted values to between 0 and 1 [10, 11]. Quasi-binomial error structures were used to account for overdispersion of the residuals. Using a stepwise reduction in terms approach the differences between models were assessed using *F*-tests at *p* ≤ 0.1. Terms that did not significantly contribute to the explanation of deviance in the model were deleted [30]. Model accuracy was assessed using Pearson’s correlation (*r*) between observed area burnt in a year and area burnt predicted by the final reduced model. The same procedure was applied within each of the four defined moisture regimes to address the second objective–to test the applicability of the global hypothesis at the local scale of the Mudumalai SDTF. Residuals from all models conformed to normality and homoscedasticity. Analysis was done in software R version 2.14.0 (R Development Core Team 2011).

## Results

### Area burnt in the landscape and within moisture regimes

On average, 20.1% (± 3.2%, 1SE) of the Mudumalai landscape burnt between 1991 and 2010; this translates into a mean interval between fires of 4.9 years. The most extensive fire occurred in 1991 with 49% of the landscape burnt, and the lowest in 2006 with only 0.1% burnt. Within the moisture regimes, the wettest regime (MR1) which corresponds to tropical moist deciduous and semi-evergreen forest and the driest regime (MR4) corresponding to dry thorn forest were the least affected by fire. The intermediate regimes (MRs 2 and 3) corresponding to degraded moist deciduous, dry deciduous and dry dipterocarp forest burnt the most. Large scale fires were a rare occurrence at the extremes of the moisture gradient, with 5.2% of MR1 and 8.9% of MR4 burnt on average across all years. On the other hand, the intermediate regimes experienced an average area burnt of 23.6% and 34.0% in MR2 and MR3 respectively (Figure A in S4 Appendix). The average proportion area burnt translates into the following mean intervals between fires: MR1–19.2 yr, MR2–4.2 yr, MR3–2.9 yr, and MR4–11.6 yr.

### Relationships between proportion area burnt and seasonal rainfall across the landscape

**Fig 1:**
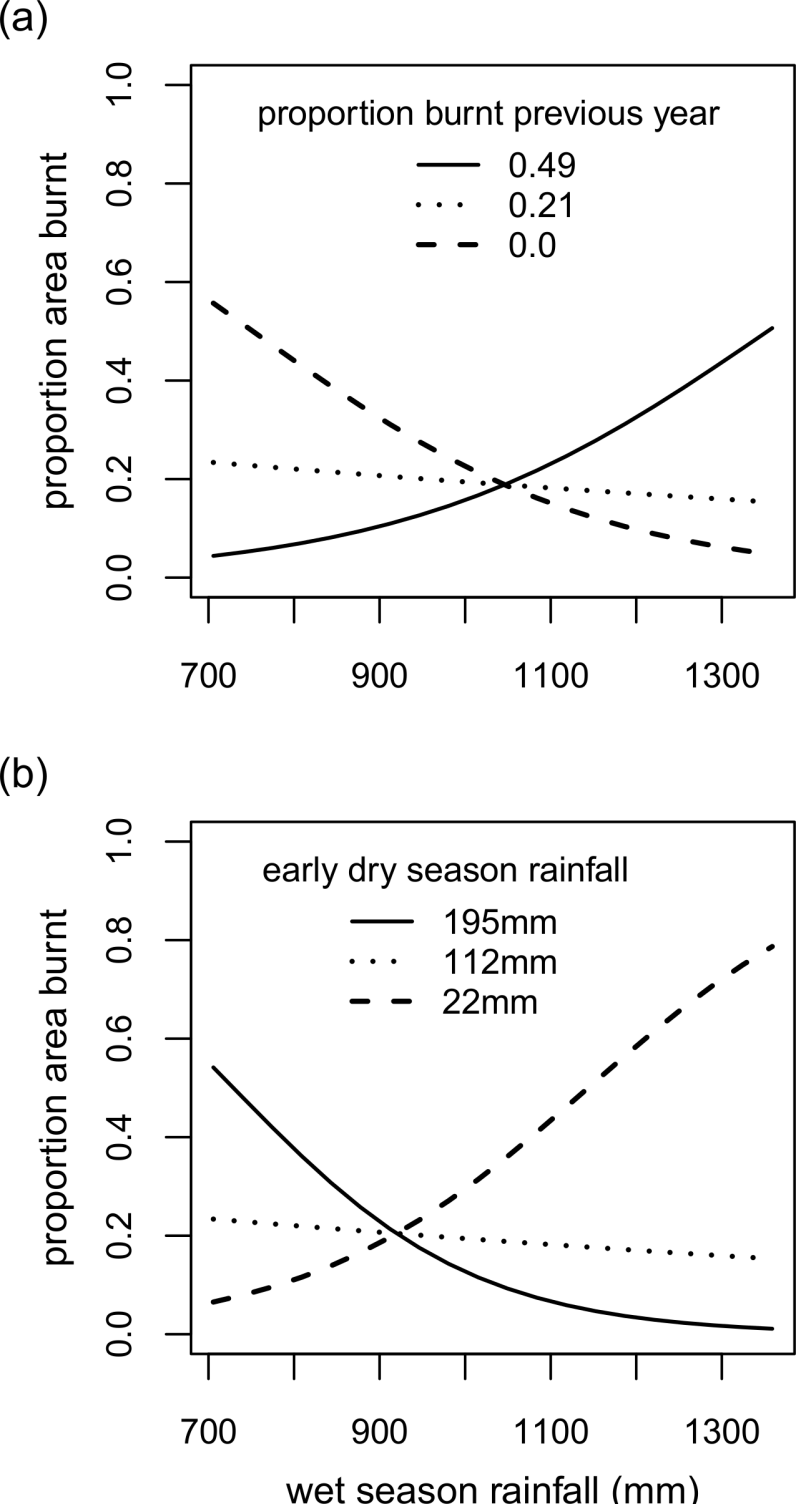
Results from the final reduced model of proportion area burnt for the Mudumalai landscape. (a) Effect of wet season rainfall on proportion area burnt in a year keeping early dry season rainfall constant at its mean value (112mm) and with previous year’s proportion area burnt held constant at three values– 0.49 (maximum of the range; solid line), 0.21 (mean of the range; dotted line) and 0.0 (minimum of the range: dashed line). (b) Proportion area burnt for a year at different values of wet season rainfall keeping previous year’s proportion area burnt constant at its mean value (0.21) and early dry season rainfall held constant at three values– 195mm (maximum of the range; solid line), 112mm (mean of the range; dotted line) and 22mm of rainfall (minimum of the range: dashed line).

In the ‘landscape model’ two 2-way interactions were significant at *p* ≤ 0.1 –one was the interaction between wet season rainfall and proportion area burnt the previous year, and the second was that between wet season rainfall and early dry season rainfall (Table 1). Proportion area burnt in a given year was estimated from the model using three values within the range used in the dataset for the variables proportion area burnt in the previous year to illustrate the first interaction (Fig 1A), and for early dry season rainfall to illustrate the second interaction (Fig 1B). According to the first interaction, when proportion area burnt the previous year was negligible, the proportion area burnt in a given year was high when wet season rainfall was low, and decreased with increasing wet season rainfall. In contrast, when c.50% of the study area had burnt the previous year, proportion area burnt in a year increased with increasing wet season rainfall (Fig 1A). According to the second interaction, in situations where early dry season rainfall was high, the proportion area burnt was high at lower values of wet season rainfall, and decreased with increase in wet season rainfall. However, when early dry season rainfall was low, the proportion area burnt increased with higher wet season rainfall (Fig 1B).

**Table 1:** Estimate of coefficients (in logit) for the terms in the final reduced model for the landscape, and their associated significance values.

	Estimate	Std. Error	t-value	Pr(>|t|)
(Intercept)	-4.121	3.593	-1.15	0.271
burn.prev.prop	-20.730	11.090	-1.87	0.083 *
wet	0.004	0.004	0.95	0.361
earlydry	0.070	0.042	1.67	0.117
burn.prev.prop: wet	0.020	0.010	1.93	0.074 *
wet: earlydry	-0.00008	0.00004	-1.82	0.090 *

Factors with ‘:’ in between represent an interaction term. burn.prev.prop–proportion area burnt in the previous year; wet–wet season rainfall of the previous year; earlydry–early dry season rainfall.

‘*’ indicates terms significant at the 0.1 level. Pearson’s correlation (*r*) between actual proportion area burnt and results from the final reduced model at the landscape level was 0.55.

### Relationships between proportion area burnt and seasonal rainfall within moisture regimes

On stepwise reduction of terms from the 3-way interaction model applied to each moisture regime, all terms except one dropped out in the wettest moisture regime MR1 (Table 2A), two 2-way interactions were significant in MR2 (Table 2B), whereas only one 2-way interaction was retained in the models for the drier MR3 and MR4 (Table 2C and 2D respectively).

**Table 2:** Estimate of coefficients (in logit) for the terms in the final reduced model for each of the moisture regimes and their associated significance values.

	Estimate	Std. Error	t-value	Pr(>|t|)
**(a) MR1: ≥1400mm**				
(Intercept)	-1.621	0.639	-2.54	0.021 **
earlydry	-0.015	0.007	-2.08	0.053 *
**(b) MR2: 1400-1200mm**				
(Intercept)	-2.217	3.635	-0.61	0.552
burn.prev.prop	-42.990	14.560	-2.95	0.011 **
wet	0.002	0.004	0.56	0.584
earlydry	0.092	0.049	1.90	0.079 *
burn.prev.prop: wet	0.035	0.012	2.92	0.011 **
wet: earlydry	-0.00009	0.00005	-1.99	0.066 *
**(c) MR3: 1200-1000mm**				
(Intercept)	-7.412	3.951	-1.88	0.079 *
wet	0.008	0.005	1.81	0.089 *
earlydry	0.073	0.038	1.92	0.073 *
wet: earlydry	-0.00008	0.00004	-2.00	0.063 *
**(d) MR4: ≤1000mm**				
(Intercept)	-19.750	4.457	-4.43	0.00042 ****
wet	0.026	0.006	4.28	0.00057 ****
earlydry	0.122	0.037	3.32	0.00433 ***
wet: earlydry	-0.0002	0.00005	-3.70	0.00195 ***

Factors with ‘:’ in between represent an interaction term. burn.prev.prop–proportion area burnt in the previous year; wet–wet season rainfall of the previous year; earlydry–early dry season rainfall.

‘*’ indicates terms significant at the 0.1 level

‘**’ - 0.05 level

‘***’ - 0.01 level

‘****’ - 0.001 level.

Pearson’s correlations (*r*) between actual proportion area burnt in a given year and results from the final reduced model for each moisture regime were as follows–MR1: 0.33; MR2: 0.83; MR3: 0.47; MR4: 0.89.

**Fig 2:**
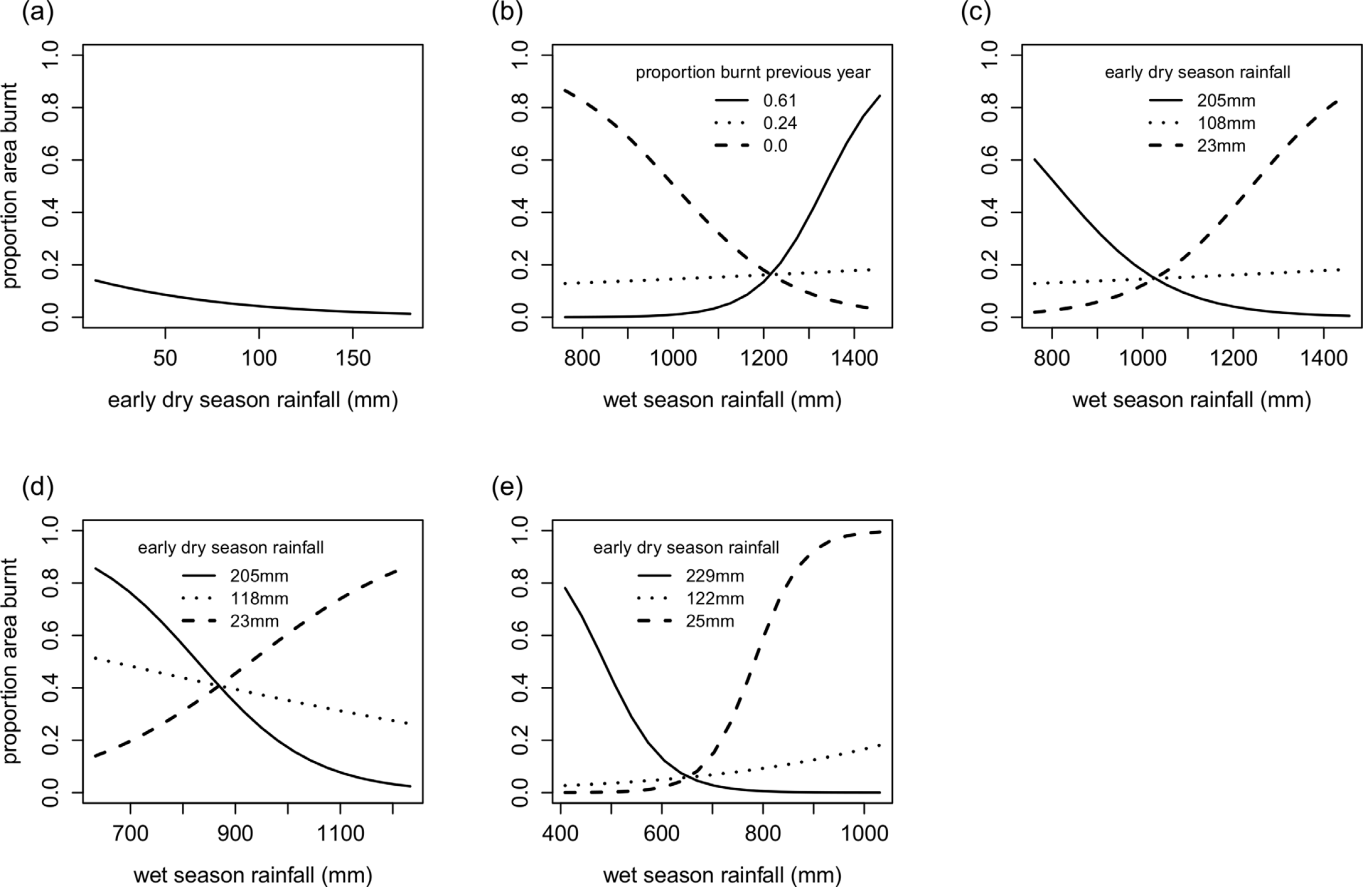
**Results from the final reduced models of proportion area burnt for each of the moisture regimes (a) MR1: ≥1400mm (b, c) MR2: 1400-1200mm (d) MR3: 1200-1000mm and (e) MR4: ≤1000mm.** The three lines in (b) represent area burnt at three levels (maximum, mean and minimum) of the range of the previous year’s proportion area burnt when early dry season rainfall is held constant at the mean (112mm) for MR2. The three lines in (c) represent area burnt at three levels (maximum, mean and minimum) of the range of early dry season rainfall when the previous year’s proportion area burnt is held constant at the mean (0.21) for MR2. The three lines in (d) and (e) represent area burnt at three levels (maximum, mean and minimum) of the range of early dry season rainfall particular to the respective regimes.

For the wettest moisture regime (MR1) the proportion area burnt decreased with increasing levels of early dry season rainfall (Fig 2A). This implies that rainfall in the two months prior to the actual fire season is critical in determining fuel moisture levels in the wettest areas. Results from the model for MR2, representing a relatively moist regime, are similar to that of the landscape model. Two 2-way interactions–wet season rainfall the previous year with proportion area burnt the previous year, and wet season rainfall the previous year with early dry season rainfall–were significant in the reduced model for this regime. Thus, a larger area burnt in situations where proportion area burnt the previous year was negligible and wet season rainfall was lower, and also when proportion area burnt the previous year was large and wet season rainfall was higher (Fig 2B). From the second interaction, a larger area burnt when early dry season rainfall was high and wet season rainfall was lower, and when early dry season rainfall was low and wet season rainfall high (Fig 2C).

Areas burnt in MR3 and the driest regime, MR4, were influenced by the same factors—wet season rainfall, early dry season rainfall and a single 2-way interaction between wet season rainfall and early dry season rainfall. According to the models, the proportion area burnt in a given year was not dependent on the proportion area burnt the previous year. In these regimes the proportion area burnt was high at lower values of early dry season rainfall when wet season rainfall the previous year was high, or at higher values of early dry season rainfall when wet season rainfall was low (Fig 2D and 2E). A notable difference between the model results for the two regimes is for situations of average early dry season rainfall. In MR3, the proportion area burnt was higher as compared to MR4 with values ranging from 0.26 to 0.51 in MR3, whereas in MR4 this ranged from 0.03 to 0.18. Thus, according to the models, in a given year the average proportion area burnt would be much higher in MR3 compared to MR4 for average levels of early dry season rainfall received.

## Discussion

In this study we tested the global “varying constraints hypothesis” of fire in terms of fuel availability and fuel moisture in natural ecosystems at two scales in a southern Indian SDTF: first, examining fire extent in the Mudumalai landscape in order to place this SDTF within the context of the hypothesis and, second, examining fire extent within different moisture regimes in Mudumalai to examine how various aspects of the varying constraints hypothesis apply to different moisture regimes across the landscape.

### Interpretation of the landscape fire model

Results from the landscape model in our study illustrate that area burnt in the Mudumalai SDTF is influenced both by fuel load and fuel moisture, thus placing this SDTF at the centre of the global gradient with regard to the “varying constraints hypothesis” of fire [7]. Fuel load in SDTFs is mainly influenced by two factors–fuels burnt and rainfall received prior to a given fire season. The area of the landscape burnt in the previous year could inhibit the spread of fire by reducing fuel amount [11], fuel moisture content [31] as well as fuel connectivity [32]. Wet season rainfall, however, influences vegetation productivity for this study area (Figure C in S2 Appendix) and, therefore, the degree to which biomass accumulates on the forest floor both from leaf fall and regeneration of understorey plants such as grasses. The interaction between area burnt and wet season rainfall in a year, thus, influences the extent burnt the following year. This can be seen from the model results in this study (Fig 1). If area burnt in a year is large, a higher amount of wet season rainfall would be required for biomass to accumulate as fuel for fires the following fire season, resulting in a larger area burnt. However, if area burnt in a year is negligible, wet season rainfall contributes less to vegetation productivity for that year, and more to influencing the degree to which the fuels are desiccated. Thus, lower wet season rainfall in situations of negligible area burnt could lead to large fires the following year.

In addition, the degree to which biomass accumulated over the wet season is dried and cured could be influenced by early dry season rainfall. The initial drying of potential fuels is dependent on early withdrawal of the monsoon, i.e. a relatively dry November-December. By January to March, the period at Mudumalai when there is little or no rain, the fuels typically dry out sufficiently for them to burn. Hence, we see that area burnt at Mudumalai is high when early dry season rainfall is low (Fig 1). The model also suggests that in some years where wet season rainfall is low, vegetation productivity could contribute to high fuel loads if early dry season rainfall is exceptionally high, a pattern that has been similarly observed in savannas of southern Africa [33].

Where would we place the Mudumalai SDTF in the spatial gradient of the “varying constraints hypothesis”? The global fire activity analysis [7] explicitly deals with biomes [34] that also comprise the global distribution of SDTFs [35] which are: “tropical/subtropical dry broadleaf forest”, “tropical/subtropical grassland, savanna and shrub”, “Mediterranean forest, woodland and scrub” and “desert/xeric shrublands”. The Mudumalai SDTF would fall under the category of “tropical dry broadleaf forest”. According to the authors [7], tropical/subtropical dry broadleaf forest would be placed more toward the “moisture limited” end of the global gradient, as monthly fire activity was found to be negatively correlated with soil moisture during the peak fire season as well as during the months immediately prior to the fire season. In contrast, fire activity in deserts, xeric shrublands, tropical/subtropical grasslands as well as savannas (to a certain extent) were inferred to be “fuel limited” as fire activity was positively correlated with soil moisture 4–8 months as well as 2 months prior to the fire season, implying the influence of increased fuel load on higher fire activity. However, our observations at the Mudumalai landscape indicate the influence of both fuel moisture and fuel load on fire extent. This highlights the difficulty in placing SDTFs as an entity in the context of the global gradient, because of the variation in forest structure for ecosystem types that comprise the definition of SDTFs. Thus, a finer scale examination of SDTFs is warranted as illustrated by our analysis of the four defined moisture regimes of the Mudumalai landscape described below.

### Interpretation of fire models for different moisture regimes

Model outputs for each of the moisture regimes examined individually clearly indicated that different sets of fuel-related factors contributed to the area burnt across these regimes.

Tree leaf litter constitutes the primary fuel load in the moister western portions of Mudumalai with grass biomass being negligible (Figure B in S2 Appendix). According to the model for the wettest regime, early dry season rainfall and, hence, the extent to which fuels are dried two months prior to the fire season, influences the extent to which fire spreads here. We had expected that a stronger influence such as deficiency in wet season rainfall would significantly influence area burnt in this regime. Fires are difficult to map precisely in areas falling within the wettest regime; ground surveys were often hindered due to logistical reasons and areas burnt are sometimes not adequately captured by satellite imagery due to high canopy cover even during the dry season, perhaps resulting in underestimates of annual area burnt. In addition, the analysis in this study examined annual fire occurrences with respect to rainfall received the preceding year. However, longer periods of moisture depletion may also be necessary for a large area to be burnt in the wettest regime. For example, during the study period the highest area burnt in the wettest regime occurred in the year 2004, at the culmination of a three-year drought (Figure A in S3 Appendix). The occurrence of large fires in this regime can be analogous to tropical rainforest that are abundant in biomass, which burns only in extreme situations of moisture depletion such as a prolonged drought [14,15]. In our study area, litter is typically abundant in the wettest part of the landscape (Figure B in S2 Appendix), implying that the occurrence and spread of fire is limited by fuel moisture.

Fire extent in the remaining three moisture regimes is, on the other hand, largely influenced by interactions between fuel load and fuel moisture. Fire extent in MR2, a slightly drier regime than MR1, is influenced by three factors–wet season rainfall, early dry season rainfall and area burnt the previous year–with interactions and interpretations similar to that for the overall landscape. Biomass of perennial grasses increased by 75 g m^-2^ over a time interval of 2 years in MR2 (Table A in S2 Appendix). Fuel was also found to increase from 2–5 t ha^-1^ to 5–10 t ha^-1^ in a 2–4 year period in a tropical savanna site in northern Australia [36]. Area burnt in this rainfall regime is influenced by fuel load (influenced by area burnt the previous year as well as wet season rainfall) and fuel moisture (influenced by early dry season rainfall, and sometimes wet season rainfall).

Area burnt in the drier regimes MR3 and MR4 are also influenced by fuel load and fuel moisture. Differences in fire proneness between MR3 and MR4 lie in the degree to which area burnt increases with an increase in wet season rainfall; MR4 shows much sharper increases in area burnt with wet season rainfall as compared to MR3. Higher amounts of rainfall are required either in the wet season or the early dry season for a sizeable area to burn in MR4, implying that the extent of fire in MR4 is more fuel limited than MR3.

The models for these two regimes suggest that the area burnt the previous year may not play a significant role in reducing fuel load the following dry season. Grass accumulation is high in MR3–177.5 g m^-2^ in 2 years, the highest amongst the four regimes (Table A in S2 Appendix)–and leaf litter accumulates over the dry season every year irrespective of whether fire happens or not. This could result in fires not being dependent on area burnt the previous year in MR3, and also occurring on a more frequent basis in this regime. In the driest regime (MR4) canopies are very open, with stunted, shrubby vegetation that has some physiognomic similarities to arid or semi-arid savannas in Africa. This would explain the similarity between our model inference for MR4 and that derived for southern African savannas, where a measure of accumulated rainfall in the wet season is the most significant factor in explaining area burnt [11]. This regime can also be compared to Californian chapparal ecosystems, where fuel loads are not influenced by area burnt the previous year [37]. The accumulation of litter is restricted to patches below shrubs and trees, and short grasses are the predominant fuel here in our study. Grass productivity would be influenced by wet season rainfall, or even early dry season rainfall if wet season rainfall the previous year is low.

The “varying constraints hypothesis” of fire in natural ecosystems [6, 7] can, thus, be demonstrated within a 43 km stretch of a tropical dry forest in southern India. Although the forest type can be classified generally as ‘SDTF’ it is important to note the range of vegetation types within this category, each with a particular moisture regime and, hence, fire regime. This highlights the importance of analysing an area with respect to its moisture regime and its variations. Other regional studies that have demonstrated similar trends have sharp differences in the rainfall received across their landscapes–approximately a range of 1500 mm for the wet-dry tropics in northern Australia [10, 38] and approximately 900–1200 mm in northwestern Argentina [8, 9]. Both these regions have a range of vegetation types to explore within the rainfall gradient–from open grassland to closed canopy forests. Mudumalai has a difference of approximately 1000 mm in average annual rainfall across the landscape, resulting in physiognomically different vegetation types. This affords comparison with the regional studies described above where unimodal relationships of fire extent with rainfall are found; fire activity is the lowest at either extremes of the rainfall gradient and highest in regions of intermediate rainfall. We find a similar pattern at Mudumalai with the highest extent of fire recorded in intermediate rainfall regimes (Figure A in S4 Appendix).

Unimodal relationships of area burnt with productivity and moisture gradients are not always found in regional studies. Mediterranean and temperate ecosystems of the Iberian Peninsula as a whole were found to be fuel limited rather than moisture limited [39], thus placing the region at the fuel limited end of a global gradient [40]. However, 8 of the 13 ecoregions used in the study belong to arid zones with annual average rainfall less than the average found over the 13 regions, even though the regions in the study fall across an average annual rainfall gradient of approximately 1000mm. In another study, conclusions from analyses of area burnt in ecoregions of western USA spanning productivity and moisture gradients were found to be consistent with the Varying Constraints Hypothesis [41]. However, a unimodal relationship was not found for area burnt with the productivity gradient (represented by actual evapotranspiration, AET). There was a unimodal relationship with water deficit. The authors attribute this pattern to the fact that the ecoregions defined in their study do not cover the full extent of variation in AET values found in the USA. These studies highlight the importance of noting the component regions used in an analysis of area burnt with moisture and fuel availability, particularly in the variability of fuel moisture and fuel load available in a dataset, while comparing regions to a global framework.

Climate variables other than precipitation, such as temperature [42, 43] and length of the dry season [11], for example, might explain the variation in our data. Also, fire is not the only factor that consumes vegetation and, hence, determines fuel loads for fires in such forests. Mudumalai and surrounding forests have high densities of large herbivorous mammals [44], with standing herbivore biomass comparable to that in African savannas [45]. Grazing by these wild herbivores as well as by large numbers of domestic cattle maintained by local people towards the eastern drier region of Mudumalai [46] would also have an influence on the grass biomass available as fuel. Burning in conjunction with grazing produce more pronounced effects in fuel load [47]. Human intervention is also another factor that influences wildfire extent [11, 33] even within protected areas where forest management intervene to fight fires. This is the case in Mudumalai where staff are specifically employed to monitor and extinguish wildfires during the dry season. Fire fighting policies have become more stringent with the declaration of Mudumalai as a Tiger Reserve in April 2007. These factors have, most likely, influenced the realised area burnt for some years in our dataset (see S3 Appendix for further discussion on area burnt data).

While data on the amount of fuel reduced due to grazing and data on the potential area burnt in human-impacted fire regimes are difficult to obtain, other climate variables such as those mentioned earlier in our discussion could have been included in our analysis, and these would have probably explained more of the variation in our data. However, the inclusion of additional variables would require more data as we are limited by the degrees of freedom available for analysis. More years of data on area burnt and the inclusion of additional variables that would serve as proxies for fuel moisture and fuel load could contribute to a more robust analysis. However, precise ground data on area burnt are difficult to procure in protected forested areas of India. Within this 20-year dataset at Mudumalai we find clear patterns of an association of area burnt with climate variables. Predictions of area burnt from the models for some of the moisture regimes closely matched the actual area burnt (see legend of Table 2).

Inferences from our detailed analysis of fire-climate relationships in the SDTF at Mudumalai could be extended to the extensive SDTFs in monsoonal south and southeast Asia that cover about 50% or more of forested area in this region [18, 48] but which have been poorly studied. The range in rainfall (700-1700mm) recorded in our study area essentially captures that range of rainfall (c. 500-1800mm) characteristic of SDTFs in the broader south and southeast Asian region. Obviously, local factors relating to forest structure, climate and human activity at these SDTFs could further alter the fire patterns we observed in this southern Indian SDTF. Long-term datasets such as those used in this study, otherwise generally rare for the understudied SDTFs [35], are required in order to decipher interactions between contributing environmental factors to fire and extent of area burnt in these important biomes.

## Supporting Information

S1 Appendix Study site information for Mudumalai Wildlife Sanctuary, Tamil Nadu, southern India. (PDF)

S2 Appendix Fuel load and its correlation with wet season rainfall in Mudumalai Wildlife Sanctuary, Tamil Nadu, southern India. (PDF)

S3 Appendix Variation in area burnt and seasonal rainfall in the landscape of Mudumalai Wildlife Sanctuary, Tamil Nadu, southern India. (PDF)

S4 Appendix Supplementary results of variation in rainfall and average area burnt in moisture regimes, and frequency distributions of proportion area burnt in the landscape as well as moisture regimes of Mudumalai Wildlife Sanctuary, Tamil Nadu, southern India. (DOCX)
