# Supplementary material for: Fires in Seasonally Dry Tropical Forest: Testing the Varying Constraints Hypothesis across a Regional Rainfall Gradient
Source: PLoS One. 2016 Jul 21;11(7):e0159691. doi: 10.1371/journal.pone.0159691 (PMC4956259; doi:10.1371/journal.pone.0159691)
Supplement: S1 Appendix — (PDF) [file pone.0159691.s001.pdf]

S1 Appendix
Study site information for Mudumalai Wildlife Sanctuary, Tamil Nadu, southern India

Figure A: Location of the study area, Mudumalai Wildlife Sanctuary, with respect to the surrounding topography of the Nilgiris (to the south-east of Mudumalai) in the Western Ghats, southern India.

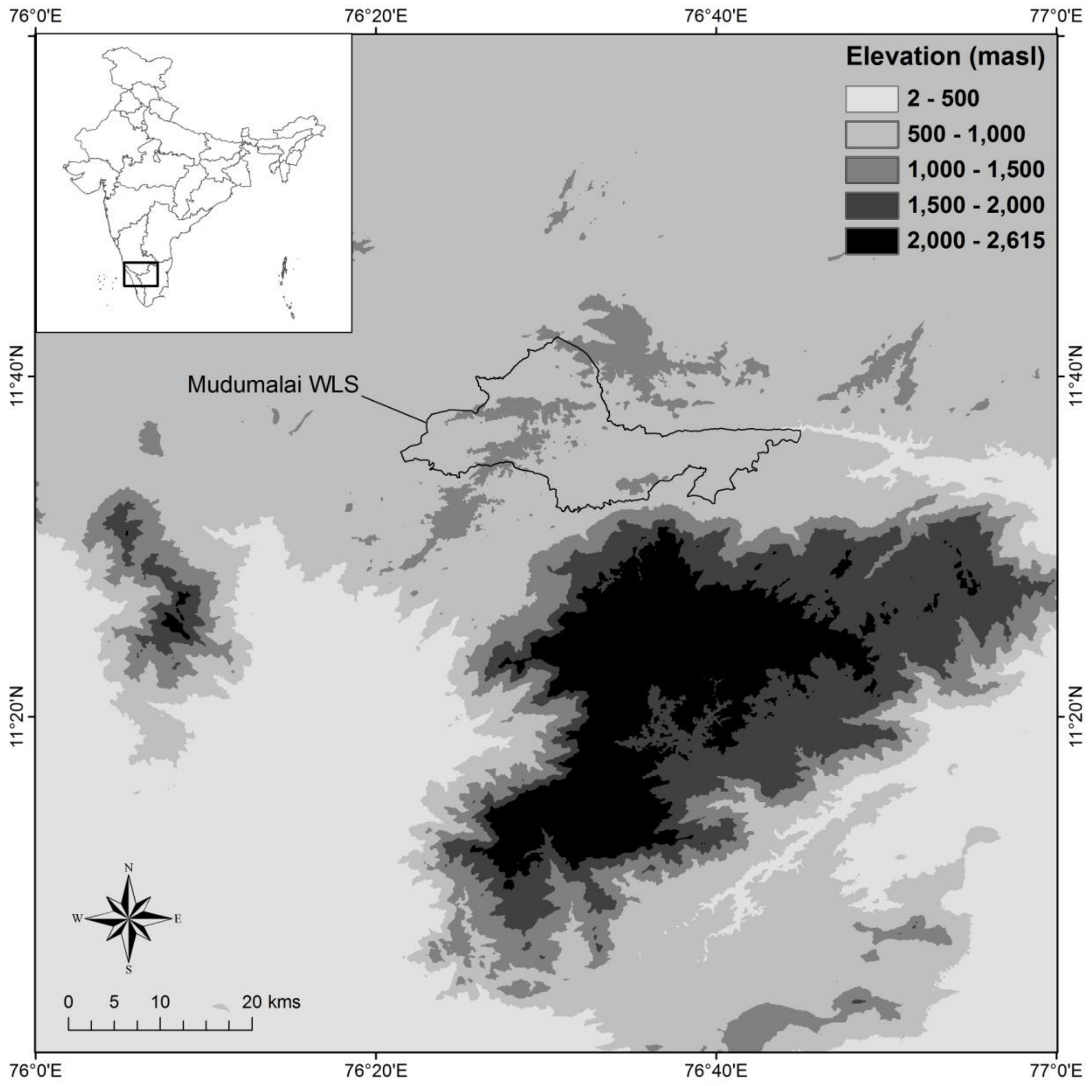

Figure B: (a) Fire frequency at Mudumalai for the time period 1990 to 2010 and (b) average annual rainfall with the four defined moisture regimes at intervals of 200mm. Both maps were produced at a 100m resolution. The fire frequency map depicts the frequency of occurrence of fire in each 100m raster cell that ranges from 0 to 13 times in the span of 20 years. The rainfall map was produced by interpolation from 13 rain gauges located in and near Mudumalai (Dattaraja *et al*. 2013).

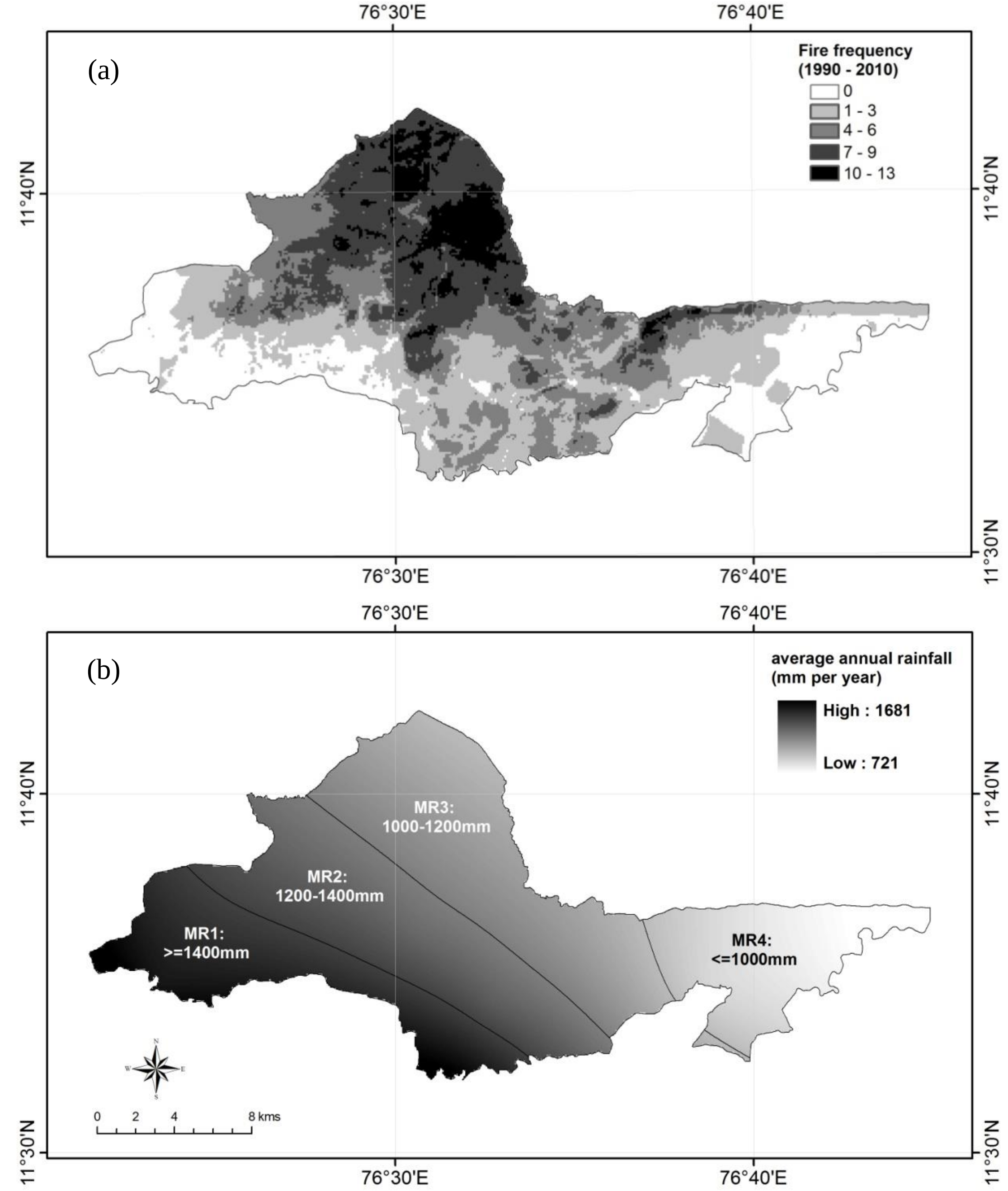

Figure C: Average rainfall per month from 13 weather stations in and around Mudumalai. Data are for the years 1991-1998. ‘West’, ‘central’ and ‘east’ refer to the approximate location of the rain gauge relative to the study area. Seasons as defined for the analysis in this study are indicated below the x-axis. The grey shaded region indicates the period when fires occur in Mudumalai.

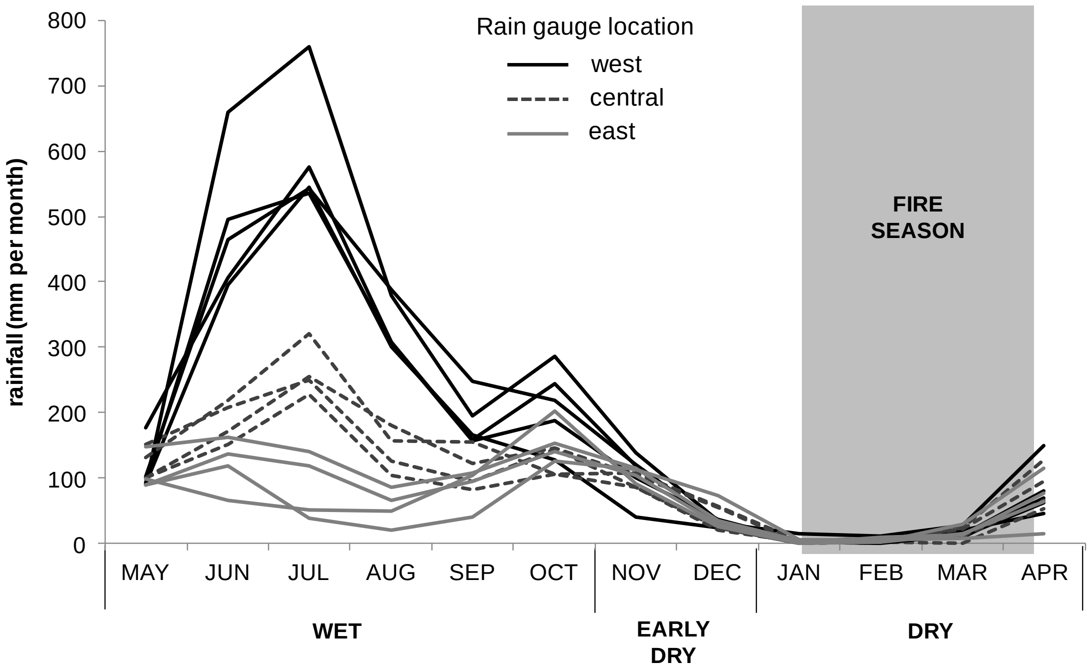

Reference:

Dattaraja, H.S., Pulla, S., Mondal, N., Suresh, H.S., Bharanaiah, C.M.B. Sukumar, R. (2013) Spatial interpolation of rainfall for Mudumalai Wildlife Sanctuary and Tiger Reserve, Tamil Nadu, India. *CES Technical Report no.130*, Centre for Ecological Sciences, Indian Institute of Science, Bangalore, India.
