# Supplementary material for: Fires in Seasonally Dry Tropical Forest: Testing the Varying Constraints Hypothesis across a Regional Rainfall Gradient
Source: PLoS One. 2016 Jul 21;11(7):e0159691. doi: 10.1371/journal.pone.0159691 (PMC4956259; doi:10.1371/journal.pone.0159691)

S2 Appendix
Fuel load and its correlation with wet season rainfall in Mudumalai Wildlife Sanctuary, Tamil Nadu, southern India

Methods

Fuel load measurement:

Initially a grid with 1.5km interval spacing was overlaid on topographic maps of the study area in a GIS. The nodes of the grid were chosen as locations for collecting biomass samples which were located in the field using a GPS. In total 87 locations were sampled across the study area over three years (Figure A). Plots were established and the first fuel load samples were collected in the months of December 2003 to February 2004. Two more samples were taken at each of the 87 locations in February 2005 and February 2006.

Grass was harvested and leaf litter collected in four 1x1m quadrats at the corners of a 20x20m plot established at each location. Leaf litter fresh weights were measured in the field using a pan-balance. The grass samples were collected in newspaper bags and air-dried till constant weight at the field station. The dry weight of grass biomass and fresh weight of leaf litter biomass were averaged over the four samples for each plot.

Figure A: **Location of sample points for fuel load measurement within Mudumalai.** The background map indicates broad forest vegetation types and the location of settlements. The rainfall contours (dashed lines) delineate the four moisture regimes within the sanctuary defined in this study (see main text) that roughly correspond to the broad forest vegetation types.

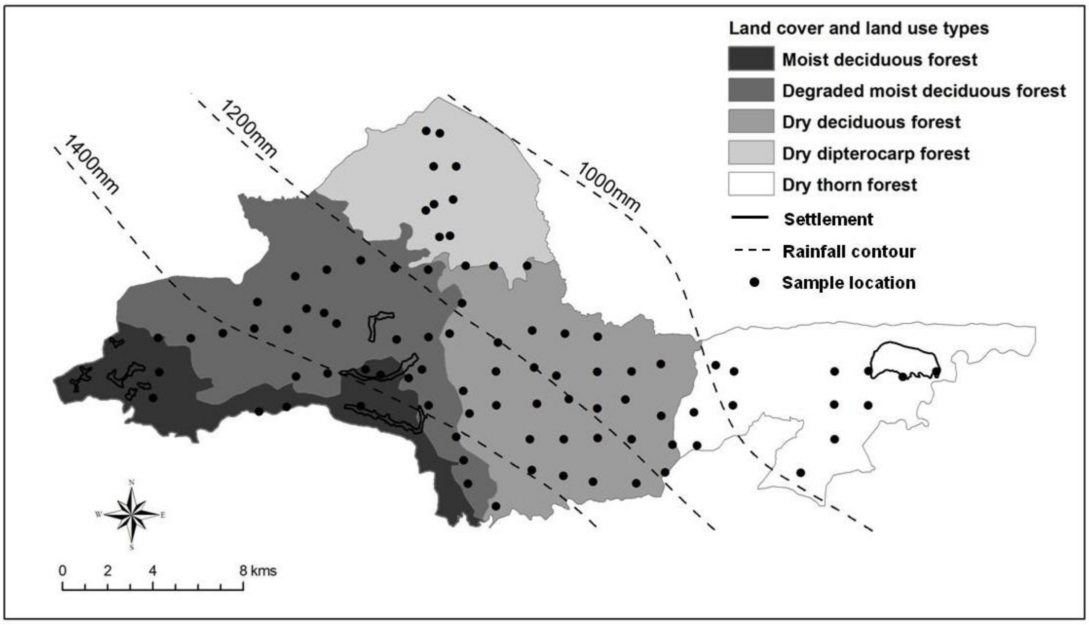

Grass accumulation

The accumulation of grass biomass over the three year period was calculated for 44 plots that had not burnt during this time. The difference between grass biomass collected in 2004 and 2006 was calculated for plots within each moisture regime (MR). Grass accumulation is also provided as a per year estimate (Table A).

Correlation of total fuel load with wet season rainfall

Total fuel load for each plot was calculated as the sum of grass dry weight and leaf litter fresh weights. Rainfall in the wet season (sum of rainfall from May to October in a year) was extracted from interpolated rainfall maps for the years 2003, 2004 and 2005 for each of the 87 point locations across the landscape. These were then correlated with the fuel load measured at each point using Pearson's correlation.

Results

Fuel load

Average fuel load was the highest in the central parts of the sanctuary (MRs 2 and 3). Grass biomass was low relative to litter biomass in the wettest regime (MR1) and driest regime (MR4) at the western and eastern extremes of the sanctuary, respectively. Average grass biomass was the highest in MR3, a region that receives intermediate levels of rainfall. Litter contributes the most to the total fuel load in MR1, the wettest regime (Figure B).

Grass accumulation

Grass accumulation was the highest in MR3, a relatively dry regime within the sanctuary, followed by MR2 (Table A).

Correlations between wet season rainfall and fuel load measurements in the field

Wet season rainfall was positively correlated with fuel load across the landscape for two out of the three years of the study (Figure C).Wet season rainfall in 2003 was positively correlated with total fuel load in 2004 ($r = 0.32$, p = 0.004), and rainfall in 2004 with total fuel load in 2005 ($r = 0.28$, p = 0.015). Rainfall in 2005 and total fuel load in 2006, however, were not correlated ($r = -0.09$, p = 0.421).

Figure B: Biomass of grass and litter averaged for sample points within each moisture regime (MR) and over the three years of sampling (2004-2006). Error bars indicate 1SE. Number of sample points (*n*) = 14 for MR1, 32 for MR2, 30 for MR3 and 11 for MR4.

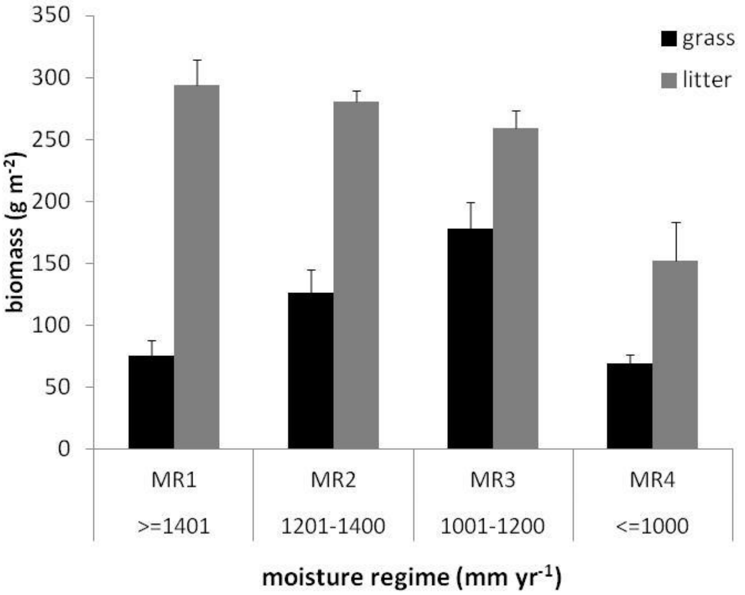

Table A: Grass accumulation between 2004 and 2006 within the four moisture regimes defined for Mudumalai.

Moisture regime (MR)	MR1 (>=1400 mm yr^{-1})	MR2 (1200-1400 mm yr^{-1})	MR3 (1000-1200 mm yr^{-1})	MR4 (<=1000 mm yr^{-1})
Grass biomass in 2004 (g m^{-2})	47.5	45.4	74.3	45.1
Grass biomass in 2006 (g m^{-2})	58.8	120.8	251.8	65.9
Difference in biomass between 2004 and 2006 (g m^{-2})	11.4	75.4	177.5	20.8
Grass biomass accumulation per year (g m^{-2} yr^{-1})	5.7	37.7	88.8	10.2
Number of plots	9	15	14	6

Figure C: Relationship between wet season rainfall and fuel load measurements in Mudumalai.

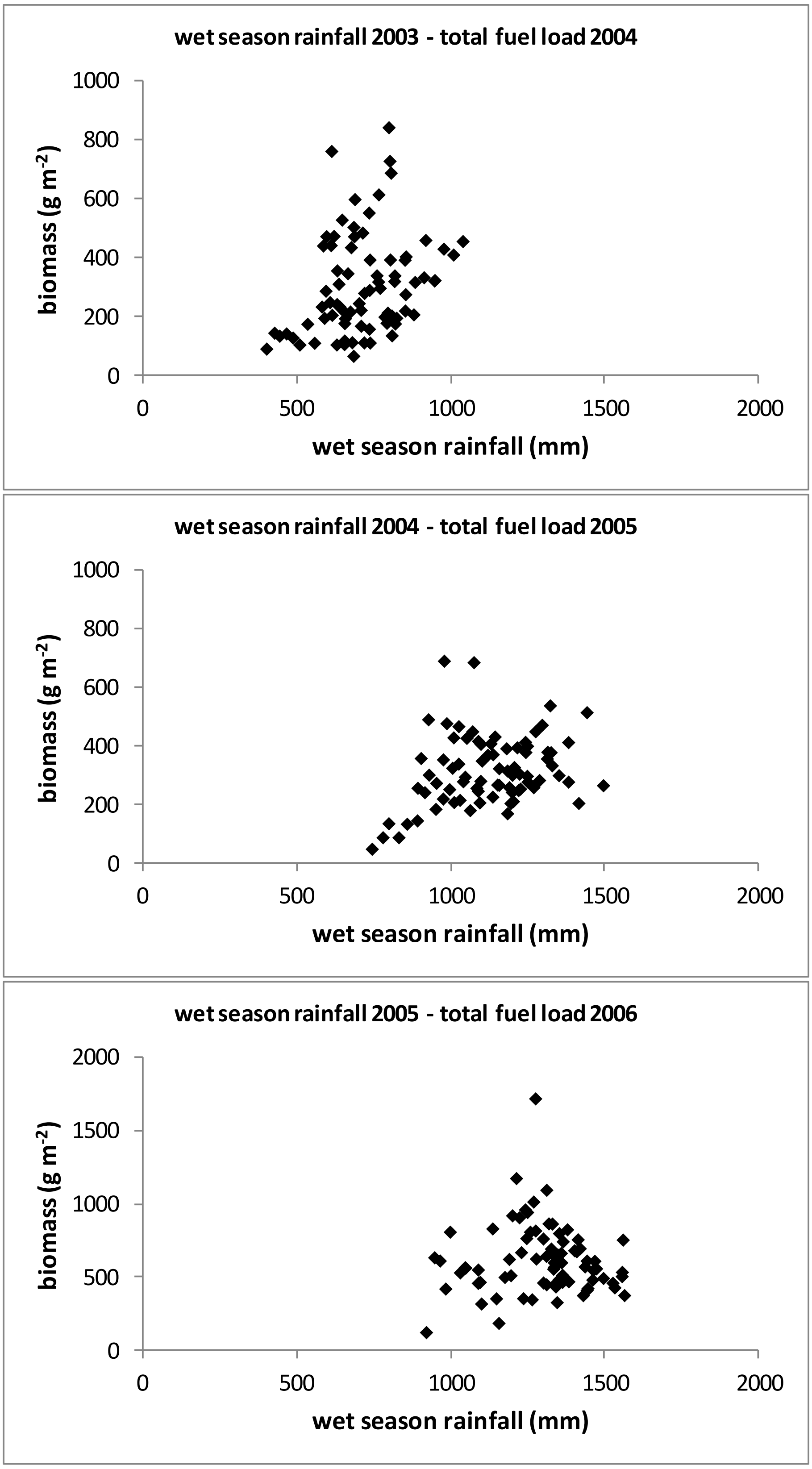

Supplement: S2 Appendix — (PDF) [file pone.0159691.s002.pdf]
