# Supplementary material for: Fires in Seasonally Dry Tropical Forest: Testing the Varying Constraints Hypothesis across a Regional Rainfall Gradient
Source: PLoS One. 2016 Jul 21;11(7):e0159691. doi: 10.1371/journal.pone.0159691 (PMC4956259; doi:10.1371/journal.pone.0159691)
Supplement: S3 Appendix — (PDF) [file pone.0159691.s003.pdf]

S3 Appendix
Variation in area burnt and seasonal rainfall in the landscape of Mudumalai Wildlife Sanctuary, Tamil Nadu, southern India

Figure A: Area burnt in a year with wet season rainfall and early dry season rainfall the previous year. Wet season is defined as the months of May to October the previous year to the fire year. Early dry season is defined as the months of November and December the year previous to the fire year. The rainfall points represent the means of the distribution of data extracted from the interpolated maps for 87 plots across Mudumalai (S2 Appendix). Note: the two x-axes representing the year of rainfall (above) and the year of fire (below) are at a one-year lag.

The spatial average of wet season (May-October) rainfall at Mudumalai ranged from 705mm in 2003 to 1359mm in 1994 for the period 1990-2009. Notable deficits (≤ 20% from the 20-yr mean) in wet season rainfall occurred between 2001 and 2003 when rainfall was 24-32% below the average for this season. Years with excess (≥ 20% from the 20-yr mean) wet season rainfall were 1992, 1994 and 2005, when Mudumalai received 21-31% higher rainfall than average. Early dry season rainfall during the same period ranged from 22mm in 2008 to 195mm in 1998.

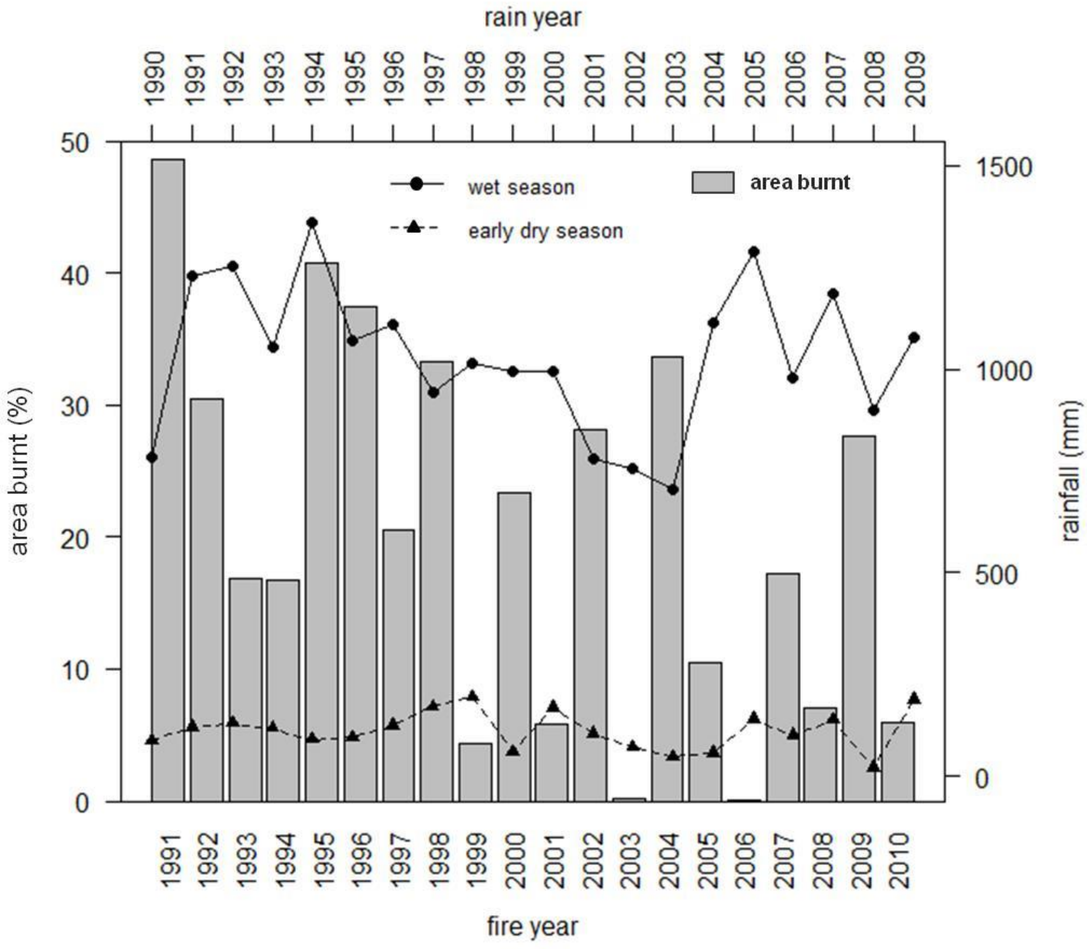

Note on area burnt data:
There appears to be a declining trend in area burnt from 1991 to 2010 in the figure above. There could be several reasons for this trend other than the influence of climate variability.

One reason could be due to differences in the manner in which area burnt had been mapped from 1989 to 2002, and then from 2003 to 2010. For the period 1989-2002 fire maps were made by surveying on the ground and marking out the same on hardcopy topography maps. The maps were then digitised from the scanned topographic sheets in a GIS software, converted to polygon data and area burnt calculated. From the period 2003-2010 area burnt has been mapped using GPS receivers. Areas that were burnt were walked along the perimeters with the GPS in track mode. The GPS tracks recorded were then converted into polygons in a GIS (ArcGIS 9.2) and area burnt calculated using UTM projection (UTM zone43, datum: WGS 84). The maps of the earlier period might, therefore, record somewhat larger areas burnt compared to the latter period. In fact, within the latter period, there are two years 2004 and 2005 where a few patches of area burnt were mapped by techniques employed in the earlier period (reasons: in 2004 the patch burnt was in an area frequented by poachers who were a security risk to the researchers, and in 2005, due to logistic constraints). Hence, because of the rough estimate of area burnt for these patches, the overall area burnt might be an over-estimate (although for the year 2005 there was a good match between the roughly mapped patch and the area burnt estimated from satellite imagery, S3 Table).

***Table A**: Area burnt for a few years calculated from two methods of estimation – ground survey data and classified satellite imagery*

Year	area burnt (km^2)		extent of overlap (percent of area mapped from ground surveys)
	ground surveys	satellite imagery classification[1]	
1996	107.4	123.4	66.5
1997	50.4	67.5	73.9
1999	9.7	14.6	14.5
2001	32.0	19.1	43.6
2002	183.1	92.3	35.3
2004	110.44	22.7	11.3
2005	34.37	34.1	75.7

[1]Satellite imagery for the years 1996, 1997, 1999, 2001, 2002, 2004 and 2005 were classified for area burnt; data from Kodandapani *et al.* 2008, Kodandapani N. pers comm.
[2]Satellite imagery classification is highly dependent on the date of acquisition of the image. Most satellite images acquired in April and May have cloud cover and, hence, area burnt cannot be estimated. Therefore, in some years area burnt would be an under-estimate because of the date of the satellite image would be prior to the period of frequent or extensive fire activity. The year 2004 is such an example, where the

satellite image used was acquired in February 2004, but most fires occurred in March that year.

Another reason for the declining temporal trend in extent of area burnt could be more active management of fires. The goal of park management is to prevent fires from spreading by either actively beating out the flames if the fire is approachable, or controlled with the use of back-fires in the case of fast-moving fires. Since the declaration of Mudumalai as a Tiger Reserve in April 2007, the park administration has been able to employ a much larger number of fire-fighting staff and formulate new policies to suppress fires.
