# Supplementary material for: Fires in Seasonally Dry Tropical Forest: Testing the Varying Constraints Hypothesis across a Regional Rainfall Gradient
Source: PLoS One. 2016 Jul 21;11(7):e0159691. doi: 10.1371/journal.pone.0159691 (PMC4956259; doi:10.1371/journal.pone.0159691)
Supplement: S4 Appendix — (DOCX) [file pone.0159691.s004.docx]

**S4 Appendix**

**Supplementary results of variation in rainfall and average area burnt in moisture regimes, and frequency distributions of proportion area burnt in the landscape as well as moisture regimes of Mudumalai Wildlife Sanctuary, Tamil Nadu, southern India**

**Figure A:** Variation in (a) wet season rainfall and (b) early dry season rainfall, and (c) average proportion area burnt (error bars indicate 1SE) in each of the four moisture regimes across the 20-year (1990-2010) study period at Mudumalai. When seasonal rainfall is compared across different moisture regimes at Mudumalai, rainfall is distinctly different between MRs 1 and 4 in the wet season but not during the early dry season.

(a)

(b)

(c)

**Figure B:** Frequency distributions of proportion area burnt in (a) the landscape and (b) in each of the moisture regimes. Frequency refers to the number of occurrences of proportion area burnt within a particular size-class. Note: y-axis ranges are different for each graph.

(a)

(b)

(b)
